# Glimpses of Ancient Physic and Physicians

**Published:** 1850-04

**Authors:** 


### GLIMPSES OF ANCIENT PHYSIC AND PHYSICIANS.

Until the introduction of medicine into Greece, little is known respecting its history; in fact, for many centuries subsequent to this period, it wears the garb of mystery and superstition. The most learned and inquisitive historians inform us that the Egyptians first cultivated the subject successfully; however, some writers ascribe the merit of its discovery to the Assyrians. Medicine and disease are, doubtless, coeval. The priests, who were the common depositaries of knowledge,* seem to have been in possession of all that was known concerning Physic, which consisted in little else than the dexterous employment of magic arts, and other ceremonies alone calcu-

* I would refer the inquisitive student to a treatise on this subject by Dr. Leclerc, of which I have made much use.

lated to impress the minds of the people with a sense of their greatness.

Chiron, the centaur, appears to have made the Greeks acquainted with the art of medicine about the thirteenth century, before the Christian era. Much mystery is attached to his character. The probability is, he was a prince of Thessaly, and must have been an individual of profound learning. He was appointed tutor to Achilles, taught Hercules in astronomy, imparted medical knowledge to the Argonauts, also to the heroes who were at the siege of Troy. Fable says he not only bestowed his art on Æsculapius, but had charge of him from his earliest infancy. With all his skill in surgery, he does not seem to have been able to cure a wound inflicted accidentally by one of Hercules arrows, which had been poisoned with the blood of the Lernæan Hydra. Such was his extreme torture in consequence of this misfortune, that he desired death, but being born of immortal parents, that wish was denied him. The gods at length translated him into the firmament, where he became a constellation, called Sagittarius.

Our medical annals furnish us with no parallel to this lofty professional altitude. This great pioneer in medical science, (if credit be due the strange art of the Chaldeans,) continued during many succeeding centuries to impart his wholesome lessons to mankind, through the medium of judicial astrology.

The merit of having cultivated medicine, as a science, and of having made it a distinct object of pursuit, is, by common consent of antiquity, awarded to Æsculapius. He made such considerable improvements, that his countrymen paid him divine honors, erected temples to him in various parts of Greece, and even deduced his birth from Apollo himself. He is supposed to have been a native of Epidaurus, where he was accidentally discovered in an exposed situation by a shepherd,* (probably in consequence of his illegitimate birth,) and placed under the protection of Chiron. Fable says Apollo destroyed

---

* The shepherd alluded to was, probably Apollo, as he is said to have fed the cattle of Admetus.

the mother of Æsculapius through motives of jealousy, and removed him from her body after she had expired. We have no distinct account of the medicines employed by Æsculapius. The manner of his death, as related in fabulous history, proves him to have been a practitioner of consummate skill. Pluto, becoming offended in consequence of the number of subjects he rescued from his grasp, persuaded Jupiter to kill him with thunder. We have reason to conclude the means he made use of were scarcely capable of making any physical impression, for we learn that he restored Hippolytus to life by the employment of simples; and, at a very much later period, it seems that Antonius Musa cured the Emperor Augustus of a dangerous malady, by the use of cold baths, a practice previously considered extremely hazardous. For this bold and successful experiment, besides many valuable and magnificent presents showered upon him by the Emperor, he, together with all other physicians, were exempted from taxes forever, (*an immunity not revoked until the nineteenth century,*) and had a statue erected to him near that of Æsculapius. We are compelled to believe, that in the times of Æsculapius, charms and incantations were depended upon almost exclusively for the cure of internal diseases, as magic made no inconsiderable part of surgical practice.

Æsculapius transmitted his art to his sons, Podalirius and Machaon, who acquired great celebrity in surgery: the former affords us the first example of heroic practice. On his return from the Trojan war, being driven by a tempest on the coast of Caria, he cured a daughter of King Damœtus by bleeding her in both arms. His patient afterwards became his wife. He had, among other children, Hippolochus, from whom Hippocrates claims to have descended.

For many centuries following the death of the sons of Æsculapius, no individual seems to have been sufficiently skilled in medicine to appear above the surface of the common multitude. The descendants of Æsculapius, denominated Asclepiadæ, (and upon whom devolved the management of the various temples erected to his honor,) were the only physicians. These edifices were built in healthy situations, and were the common receptacles of the sick. Here, amidst disease of every grade and variety, where morbid action was daily witnessed in all its phases, and the opportunity of observing the effect of various agents on the body so abundant, how is it possible for men of judgment and sagacity not to have advanced the science of medicine, could their minds have been divested of superstitious folly, and have been influenced by the dictates of sound philosophy. It was a custom for such persons as were cured, to make a memorandum of their disease, and of the means employed for their restoration. These records were as so many lamps along the pathway which the Father of medicine was destined to tread.

The first celebrated name associated with medicine, after the immediate descendants of Æsculapius, is that of Pythagoras. He lived about the sixth century before the Christian era; is supposed to have been a native of Samos, son of Mnesarchus, the sculptor, and disciple of Pherecides. He traveled into Egypt, where he remained many years; he also visited the Chaldeans, to acquire the knowledge of the Magi. After having enriched his mind with learning of various kinds, he established a school at Crotona, where the whole circle of natural knowledge was taught. Here, to a large assemblage of students from all the civilized parts of Greece and Italy, was first inculcated the principles of Anatomy, Physiology, Pathology, Ætiology, and Semeiology.

Not only did this superior genius succeed in extricating our science from the net of superstition and bigotry, and in pointing out its just relation to the genuine principles of philosophy, but he must have contributed vastly to prepare the way for that pre-eminently great man, who was destined to create an era in the practice of medicine. The interval from the time of Pythagoras to that of Hippocrates, furnishes us with the names of several celebrated individuals, whose attention was divided between Physic and other branches of natural knowledge. DEMOCRITUS so far disregarded the prejudices of his age, as to venture upon the dissection of the human body.

Herophiylus, at a somewhat later period, experimented on the living bodies of condemned criminals. Democrates acquired much reputation for his medical skill. He reduced the os calcis of King Darius, dislocated in consequence of a fall from his horse, for which he was made immensely rich. Herodicus is celebrated as the founder of gymnastic medicine, but particularly as the preceptor of Hippocrates.

After groping in almost total darkness for many centuries, an occasional light flitting, as it were, across our mysterious and benighted pathway, now, for the first time, appears a bright and fixed luminary to guide our footsteps. We mean Hippocrates, whose profound ability and brilliant success, acquired for him the title of *Father of Medicine.* He lived about the fourth century before the Christian era; was brought up among the Asclepiadæ, attached to the temple of Cos, his native place, consequently had an opportunity, from his earliest years, of observing disease, and must have possessed a mind peculiarly fitted for the pursuit of the science he embraced. So far was he in advance of his age, that his admirers discover a wild enthusiasm in their estimation of his attainments; some pretending he was deeply versed in anatomy; others go so far as to make him acquainted with the circulation of the blood. But, as comparative anatomy was little cultivated until the time of Pythagoras, and the dissection of human body regarded with so much abhorrence as to be scarcely attempted, even by the most zealous and daring, his knowledge of animal structure must have been imperfect. These considerations increase our admiration for his skill, especially when we remember the prevailing ignorance on the subjects of physiology and materia medica.

Hippocrates wrote a number of books, and his name was attached to many spurious productions; in fact, learned men were engaged a great while in attempting to separate the genuine from the suppositions. How far they have succeeded, we cannot venture to say; certain it is, the number has been very materially reduced. He believed the fluids were the primary seats of disease, whence sprung the humoral pathology, the commonly received doctrine until within a late period. His attention was attracted to that peculiar principle of living bodies which presides over and superintends its actions, promotes beneficial changes, and opposes such movements as promise to result injuriously. This principle he named "Nature." He also first remarked the tendency of particular diseases to self-cure, as a consequence of a certain train of natural actions commonly terminating in a flux from one or more of the secretory organs, but occasionally consisting in the establishment of local inflammation in some external part, progressing, in most instances, to the formation of abscess. The periods of these various vital movements, he called "*crises*," and their results, critical evacuations, inflammations, and abscesses, respectively. The importance of preserving the detail of individual cases of disease was fully appreciated by him; he paid great attention to the regulation of diet, nor was the influence of temperature, certain epidemical and other constitutions of the atmosphere, of season, locality, climate, and other analogous circumstances, beyond the reach of his penetrating genius. He investigated the forces of nature with peculiar interest, and formed a most exalted estimate of its resources; for he depended on the *vis medicatrix naturæ* for the cure of most diseases. When, from the violence or intractable nature of any distemper, he was compelled to have recourse to remedial means, the rules of prudence were never transgressed. Impressed with the belief that disease depended upon the prevalence of some morbid humors, his medicines belonged principally to the evacuant class, and especially was he partial to purgatives; he used sudorifics and diuretics; drew blood with the lancet; abstracted it topically by means of cupping-glasses; employed certain irritants, and a great variety of ointments, liniments, &c. His great capacity, as a medical practitioner, appeared in the strongest light during the Peloponnesian war, when the plague made such havoc at Athens and through Attica. He does not, however, boast of more success than commonly attends practitioners of the present day. Of forty-two patients spoken of in his first and third books on Epidemical Diseases, he owns that seventeen only were cured. Thus he indicates his candor and probity to be equal with his skill. He did not escape those feelings of mortification, caused by the injustice of a certain class of persons who impugn the skill of the physician because of ill success, forgetting, says he, "that the death of the patient is attributable to the insurmountable violence of the distemper, as much, or rather more, in most cases, than to the fault of the physician." He declared it no dishonor to solicit a consultation in certain difficult cases, so that this custom is as old as Hippocrates.

